# A Manual of Operative Surgery

**Published:** 1878-09

**Authors:** 


*A Manual of Operative Surgery.* By Lewis A. Stimson, B. A., (Yale), M. D., Surgeon to the Presbyterian Hospital, Professor of Pathological Anatomy in the Medical Faculty of the University of New York. Philadelphia: H. C. Lea. 12mo. pp. 477. With Three Hundred and Thirty-two Illustrations.

This is an excellent Manual of Surgical operations, describing in brief the methods now in practice by the most distinguished and best operators. It is a desirable work to have, because it presents quite a number of methods of operations by foreign surgeons with illustrations, that have not been given in our larger works upon surgery. The descriptions are very clear and concise and the recommendations are generally very good where they are given though the reader is for the most part left to his own judgment in choice of method. One of the best sections of the book is that which treats of plastic operations upon the face and eyelids. The subjects of excisions and amputations are very fully presented and well accompanied by illustrations. Ollier's and Langenbeck's operations upon the superior maxilla and for nasal polypus, Volkmann's excision of the rectum, operations upon the vagina and the cervix uteri are well described. The author has succeeded admirably in his purpose to present a manual of the different surgical operations now in vogue, and his work will have a large sale. It says very little on the advisability, or choice of methods of operation, and is therefore not a substitute for, but a good accompaniment to other systematic works.

